# Supplementary material for: Optimization of the Electrospray Process to Produce Lignin Nanoparticles for PLA-Based Food Packaging
Source: Polymers (Basel). 2023 Jul 7;15(13):2973. doi: 10.3390/polym15132973 (PMC10346814; doi:10.3390/polym15132973)
Supplement: Supplementary file 1 [file polymers-15-02973-s001.zip › polymers-2463894-supplementary.pdf]

Supplementary Material: Optimization of the Electrospray Process to Produce Lignin Nanoparticles for PLA-based Food Packaging

Rodrigue Daassi, Kalvin Durand, Denis Rodrigue and Tatjana Stevanovic

Table S1. Range and levels of independent process variables used in the experimental design.

Trial	Bloc	Lignin concentration	Flow rate	Applied voltage	Tip-to-collector distance
1	1	0	0	0	0
2	1	-1	1	0	0
3	1	-1	0	0	1
4	1	0	-1	0	1
5	1	0	1	0	1
6	1	0	0	1	-1
7	1	1	0	0	-1
8	1	0	-1	0	-1
9	1	-1	0	-1	0
10	1	1	0	1	0
11	1	0	-1	-1	0
12	1	1	1	0	0
13	1	1	0	-1	0
14	1	1	-1	0	0
15	1	0	0	0	0
16	1	0	-1	1	0
17	1	-1	0	1	0
18	1	0	1	0	-1
19	1	0	1	-1	0
20	1	0	0	-1	1
21	1	1	0	0	1
22	1	0	0	-1	-1
23	1	-1	0	0	-1
24	1	-1	-1	0	0
25	1	0	0	0	0
26	1	0	0	1	1
27	1	0	1	1	0
28	2	0	0	0	0
29	2	0	1	-1	0
30	2	-1	0	1	0
31	2	-1	0	0	-1
32	2	0	-1	-1	0
33	2	0	0	0	0
34	2	0	1	0	-1
35	2	0	0	-1	-1
36	2	-1	-1	0	0
37	2	0	0	-1	1
38	2	1	0	0	1
39	2	0	1	0	1
40	2	0	0	1	1
41	2	1	0	-1	0
42	2	0	-1	1	0
43	2	0	1	1	0
44	2	-1	1	0	0
45	2	1	1	0	0

46	2	0	-1	0	-1
47	2	1	-1	0	0
48	2	0	0	0	0
49	2	1	0	0	-1
50	2	1	0	1	0
51	2	0	-1	0	1
52	2	0	0	1	-1
53	2	-1	0	0	1
54	2	-1	0	-1	0

Table S2. Condition design using the response surface methodology with the Box−Behnken design and experimental/predicted data of the response.

**Run**	**TypePt**	**Bloc**	**LC (mg/mL)**	**FR (mL/h)**	**HV (kV)**	TCD (cm)	Z-Ave (d. nm)		PdI		ZP (mV)	
							EXP	PRED	EXP	PRED	EXP	PRED
**1**	**0**	**1**	**27.5**	**0.75**	**20**	17.5	250.0	212.3	0.278	0.363	-31.1	-30.9
2	2	1	5	1	20	17.5	478.6	548.5	0.504	0.446	-33.6	-32.2
3	2	1	5	0.75	20	25	667.2	668.6	0.497	0.548	-14.8	-25.3
4	2	1	27.5	0.5	20	25	462.4	381.4	0.451	0.324	-21.4	-27.4
5	2	1	27.5	1	20	25	577.2	596.1	0.488	0.655	-30.3	-29.9
6	2	1	27.5	0.75	30	10	455.4	567.2	0.289	0.515	-26.0	-30.2
7	2	1	50	0.75	20	10	384.9	518.3	0.639	0.508	-29.5	-25.2
8	2	1	27.5	0.5	20	10	352.2	256.5	0.63	0.546	-28.4	-30.8
9	2	1	5	0.75	10	17.5	636.4	477.4	0.383	0.426	-34.6	-38.1
10	2	1	50	0.75	30	17.5	490.7	431.9	0.431	0.442	-29.9	-33.7
11	2	1	27.5	0.5	10	17.5	278.2	319.0	0.517	0.420	-30.0	-32.4
12	2	1	50	1	20	17.5	839.8	648.3	0.751	0.559	-28.9	-28.3
13	2	1	50	0.75	10	17.5	712.6	639.8	0.218	0.441	-22.4	-24.8
14	2	1	50	0.5	20	17.5	218.2	271.6	0.203	0.303	-28.1	-28.9
15	0	1	27.5	0.75	20	17.5	173.1	212.3	0.226	0.363	-33.7	-30.9
16	2	1	27.5	0.5	30	17.5	334.4	330.7	0.406	0.433	-31.9	-26.2
17	2	1	5	0.75	30	17.5	723.4	681.7	0.693	0.532	-32.0	-24.9
18	2	1	27.5	1	20	10	487.3	507.9	0.213	0.428	-29.2	-30.6
19	2	1	27.5	1	10	17.5	512.7	565.6	0.651	0.485	-23.5	-29.6
20	2	1	27.5	0.75	10	25	767.2	675.6	0.537	0.464	-33.1	-30.3
21	2	1	50	0.75	20	25	522.6	541.5	0.498	0.392	-33.7	-33.0
22	2	1	27.5	0.75	10	10	367.5	349.0	0.414	0.481	-32.5	-32.7
23	2	1	5	0.75	20	10	409.4	478.7	0.401	0.428	-48.4	-37.3
24	2	1	5	0.5	20	17.5	334.4	458.9	0.359	0.491	-31.1	-29.4
25	0	1	27.5	0.75	20	17.5	134.2	212.3	0.860	0.363	-32.8	-30.9
26	2	1	27.5	0.75	30	25	438.5	453.7	0.528	0.537	-29.0	-28.4
27	2	1	27.5	1	30	17.5	700.3	550.3	0.591	0.580	-34.9	-31.4
28	0	2	27.5	0.75	20	17.5	146.7	212.3	0.182	0.363	-31.7	-30.9
29	2	2	27.5	1	10	17.5	549.5	565.6	0.526	0.485	-31.8	-29.6
30	2	2	5	0.75	30	17.5	857.3	681.7	0.703	0.532	-9.03	-24.9
31	2	2	5	0.75	20	10	674.6	478.7	0.517	0.428	-31.3	-37.3
32	2	2	27.5	0.5	10	17.5	212.3	319.0	0.515	0.420	-29.8	-32.4
33	0	2	27.5	0.75	20	17.5	462.3	212.3	0.343	0.363	-24.9	-30.9
34	2	2	27.5	1	20	10	484.1	507.9	0.375	0.428	-29.4	-30.6
35	2	2	27.5	0.75	10	10	426.7	349.0	0.478	0.481	-31.6	-32.7
36	2	2	5	0.5	20	17.5	305.6	458.9	0.305	0.491	-30.1	-29.4
37	2	2	27.5	0.75	10	25	766.7	675.6	0.496	0.464	-35.4	-30.3
38	2	2	50	0.75	20	25	383.7	541.5	0.319	0.392	-29.8	-33.0
39	2	2	27.5	1	20	25	564.2	596.1	0.590	0.655	-23.7	-29.9
40	2	2	27.5	0.75	30	25	295.4	453.7	0.542	0.537	-31.8	-28.4
41	2	2	50	0.75	10	17.5	469.9	639.8	0.295	0.441	-29.6	-24.8
42	2	2	27.5	0.5	30	17.5	346.2	330.7	0.346	0.433	-27.0	-26.2
43	2	2	27.5	1	30	17.5	497.0	550.3	0.471	0.580	-35.4	-31.4

44	2	2	5	1	20	17.5	473.1	548.5	0.505	0.446	-34.0	-32.2
45	2	2	50	1	20	17.5	657.4	648.3	0.612	0.559	-28.0	-28.3
46	2	2	27.5	0.5	20	10	331.5	256.5	0.655	0.546	-32.6	-30.8
47	2	2	50	0.5	20	17.5	393.1	271.6	0.213	0.303	-29.3	-28.9
48	0	2	27.5	0.75	20	17.5	103.6	212.3	0.277	0.363	-30.8	-30.9
49	2	2	50	0.75	20	10	555.4	518.3	0.615	0.508	-24.9	-25.2
50	2	2	50	0.75	30	17.5	466.6	431.9	0.494	0.442	-32.6	-33.7
51	2	2	27.5	0.5	20	25	464.8	381.4	0.425	0.324	-29.7	-27.4
52	2	2	27.5	0.75	30	10	418.8	567.2	0.562	0.515	-29.3	-30.2
53	2	2	5	0.75	20	25	715.9	668.6	0.455	0.548	-34.3	-25.3
54	2	2	5	0.75	10	17.5	344.9	477.4	0.391	0.426	-40.3	-38.1

LC: lignin concentration, FR: flow rate, HV: applied voltage, EXP: experimental, PRED: predicted, TCD: tip-to-collector distance, d: Sizes of nanoparticles, PdI: polydispersity index, ZP: Zeta potential.

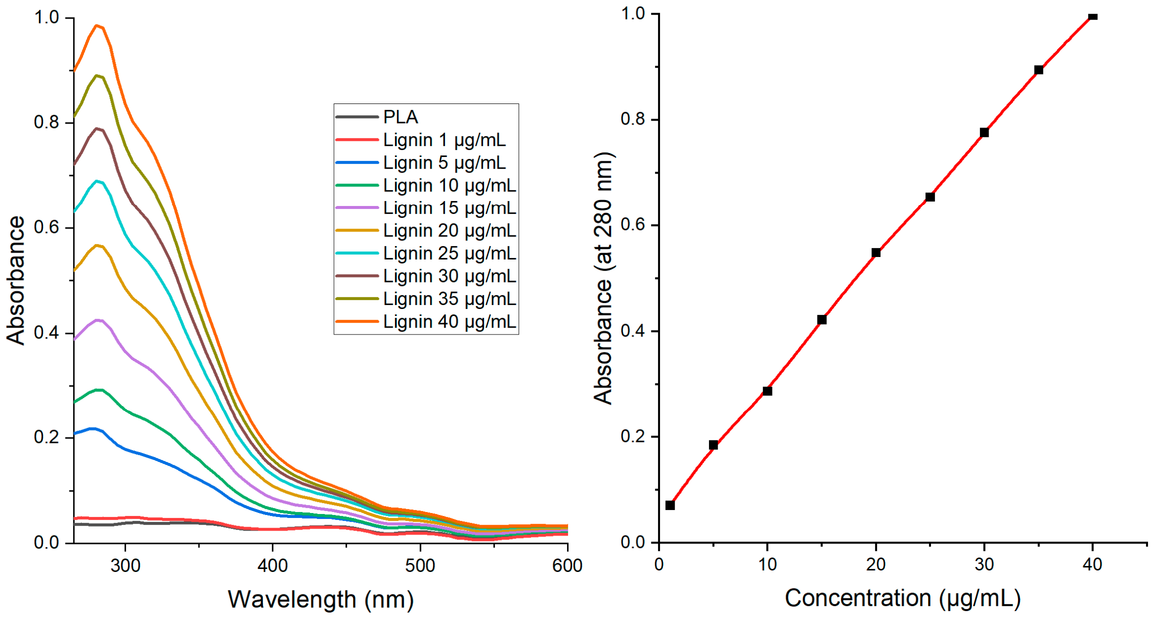

Figure S1. UV-Visible absorbance spectra of lignin nanoparticles in ethanol solutions at different concentrations (left) and calibration curve obtained by plotting the absorbance at 280 nm as a function of lignin concentration.

Table S3. Initial thermal degradation temperature (°C) and glass transition temperature (°C). The lignin content is 10 wt.%.

**Sample**	$T_{5\%}$	T_g
Lignin	-	164
Lignin nanoparticles	-	165
PLA	378.7	64.2
Lignin-PLA	374.8	62.7
LNPs-PLA	368.0	63.2
LNPs-PLA/PLA	379.3	62.7

$T_{5\%}$: Temperature at which the mass loss of the sample is 5% less than its mass loss at 50 °C via TGA; T_g: glass transition temperature determined by DSC.

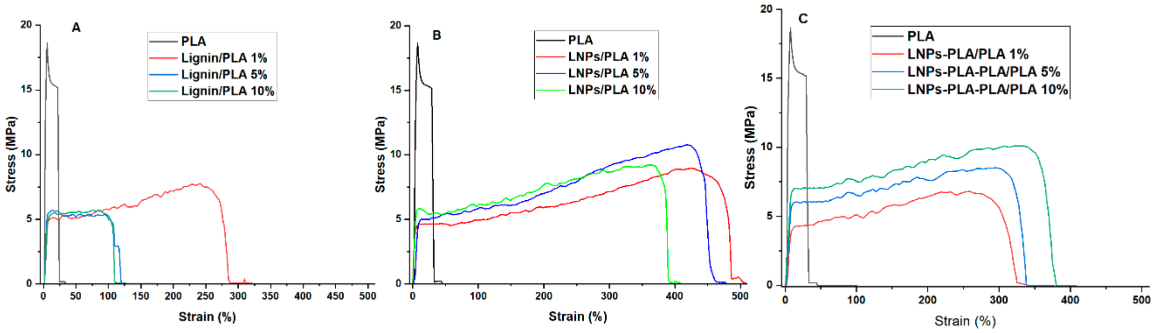

Figure S2. Stress-strain plots for neat PLA and PLA blend films containing different concentrations of lignin (A); lignin nanoparticles (B), and PLA-grafted lignin nanoparticles (C).

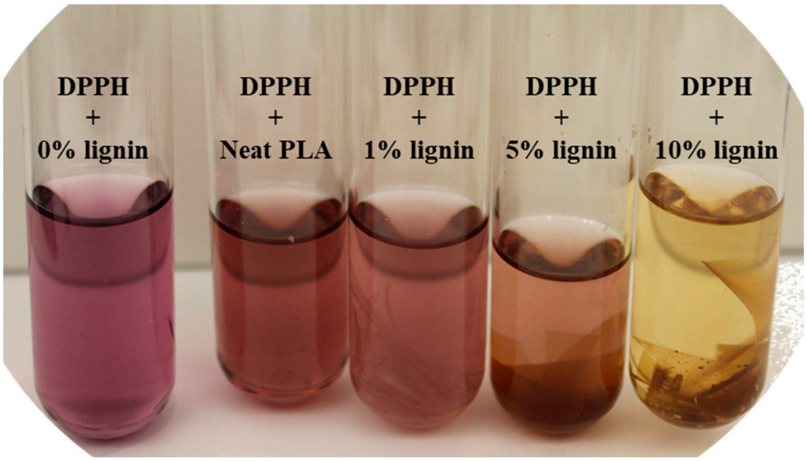

Figure S3. Image of DPPH solution and PLA composite films containing lignin at different concentrations (0-10%) immersed in DPPH solution (25% in methanol) for 4 h. The films are cut into small pieces before placing them into a DPPH solution.   .

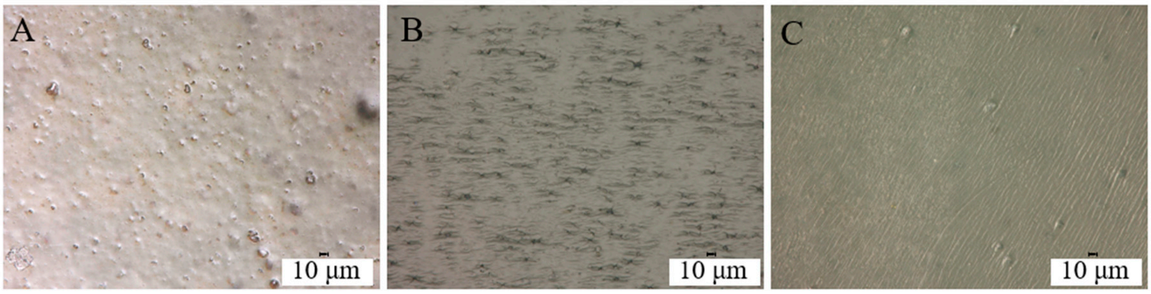

Figure S4. Digital images (thick films) of: (A) lignin-PLA blend, (B) lignin nanoparticle- PLA blend and (C) PLA–PLA-grafted lignin nanoparticle blend. All the blends contain 10 wt.% of lignin.
